# Talar trochlear morphology may not be a good skeletal indicator of locomotor behavior in humans and great apes

**DOI:** 10.1038/s41598-021-03429-y

**Published:** 2021-12-15

**Authors:** Shuhei Nozaki, Motoharu Oishi, Naomichi Ogihara

**Affiliations:** 1 Laboratory of Human Evolutionary Biomechanics, Department of Biological Sciences, Graduate School of Science, The University of Tokyo, 7-3-1 Hongo, Bunkyo, Tokyo, 113-0033 Japan; 2 Laboratory of Anatomy, School of Veterinary Medicine, Azabu University, Sagamihara, Kanagawa 252-5201 Japan

**Keywords:** Biological anthropology, Bone

## Abstract

To reconstruct locomotor behaviors of fossil hominins and understand the evolution of bipedal locomotion in the human lineage, it is important to clarify the functional morphology of the talar trochlea in humans and extant great apes. Therefore, the present study aimed to investigate the interspecific-differences of the talar trochlear morphology among humans, chimpanzees, gorillas, and orangutans by means of cone frustum approximation to calculate an apical angle and geometric morphometrics for detailed variability in the shape of the talar trochlea. The apical angles in gorillas and orangutans were significantly greater than those in humans and chimpanzees, but no statistical difference was observed between humans and chimpanzees, indicating that the apical angle did not necessarily correspond with the degree of arboreality in hominoids. The geometric morphometrics revealed clear interspecific differences in the trochlear morphology, but no clear association between the morphological characteristics of the trochlea and locomotor behavior was observed. The morphology of the trochlea may not be a distinct skeletal correlate of locomotor behavior, possibly because the morphology is determined not only by locomotor behavior, but also by other factors such as phylogeny and body size.

## Introduction

Reconstructing the locomotor behaviors of early hominids from their fossilized skeletal remains is vital to clarify the origin and evolution of human obligatory bipedal locomotion. Among all the skeletal elements, the human talus, or ankle bone, is known to be highly specialized to adapt to bipedal locomotion in the course of human evolution, as the foot most directly interacts with the ground or tree substrates during locomotion. Specifically, the morphology of the talar trochlea imposes the axis of rotation and the range of motion of the foot with respect to the tibia^1,2^. Clarifying the pattern of the morphological variations of the talar trochlea in humans and non-human great apes and how it possibly corresponds to the differences in their locomotor behavior is therefore of particular importance in reconstructing locomotor behaviors of fossil hominins and understanding the evolution of bipedal locomotion in the human lineage^2–5^.

It has been suggested that the articular surface of the talar trochlea in humans is shaped like the surface of the frustum of a cone with the apex oriented medially (but not like the surface of a cylinder), as the radius of curvature of the medial side of the trochlea is smaller than the lateral side^1,2^. Owing to the conical shape of the trochlea, the axis of rotation of the foot with respect to the tibia is considered to be inclined medially so that the anterior foot is naturally abducted and inverted during ankle dorsiflexion, and vice versa during plantarflexion^1^. In great apes, the apex angle of the conical trochlea was reportedly much larger than that of humans^2^, indicating that such natural concomitant abduction and inversion of the ankle joint due to dorsiflexion is larger in great apes^3^. Larger abduction and inversion of the foot during ankle dorsiflexion allows the plantar surface of the feet to be placed parallel to the arboreal substrate (tree trunk) for firm gripping^2,6^. Therefore, the larger apex angle of the cone-shaped trochlea in great apes is considered an adaptation to vertical climbing and arboreal locomotion^3^.

Previous studies have quantified the talar angle (or talar axis angle) to estimate the apex angle of the talar trochlea to infer the axis of rotation of the ankle joint and the degree of arboreality^2,3,7^. The talar angle is defined as the angle on the coronal plane formed by a line representing the superior surface of the talar trochlea and a line connecting the inferior-most points of the tibial and fibular facets that reportedly approximates the axis of rotation of the ankle joint^1–3^. However, the surface of the cone frustum and the axis of rotation of the ankle joint are essentially three-dimensional (3D), and the apical angle cannot necessarily be estimated two-dimensionally in the coronal plane. Therefore, the talar angle may have underestimated or overestimated the true apical angle. If the articular surface of the talar trochlea was three-dimensionally fitted by the surface of the frustum of a cone, the true apical angle of the trochlear surface could be quantified and compared, possibly providing useful morphological correlates of talocrural mobility and locomotor behavior. However, no studies have attempted to mathematically approximate the talar trochlea by a conical surface to three-dimensionally quantify the apex angle of the cone frustum, although the trochlea surface has been previously approximated by a plane^8,9^, cylinder^9^, and paraboloids^10^ for morphological analyses of the articular surface. Inman^1^ carried out the only study to measure the apex angle of human tali by physically placing a metal rod through the hole of the ankle axis and metal wires on the trochlear surface such that they intersected with the rod; however, it was not an elaborate approximation of a surface by minimizing the sum of the squared distances between a set of points comprising the talar trochlea and the surface of the cone.

The present study aimed to investigate the apical angle of the talar trochlea of human and great ape tali by means of cone frustum approximation. Specifically, we investigated whether the talar trochlea can be approximated by the cone surface, and if there was a possible correspondence between the apex angle and differences in locomotor behavior and the degree of arboreality among humans and great apes. In addition, since the cone frustum is a simple approximation of the talar trochlea, we also conducted a geometric morphometric analysis of the trochlear morphology, namely the medial and lateral rims and the central groove of the trochlea, to extract possible correlates of talocrural shape and locomotor behavior. Previous studies investigated the morphological variations of the talus among humans and great apes^2,3,5,8,11–14^. However, these studies compared the overall talar morphology but did not analyze the detailed trochlear morphology. Sorrentino et al.^5^ has recently analyzed detailed morphological variations of all the talar articular surfaces including that of the trochlea using geometric morphometrics and demonstrated that the humans possessed mediolaterally wider, flatter, and more squared talar trochlea than chimpanzees and gorillas. However, the study did not investigate the apical angle of the talar trochlea. In addition, the orangutan tali were not included in their analysis since the aim was to identify the overall morphological affinity of the fossil tali in the tali of humans and African great apes, but not to extract possible morphological correspondence between trochlear morphology and locomotor behavior.

## Materials and methods

### Sample

Computed tomography (CT) scans of tali from 20 humans, 20 chimpanzees (10 *Pan troglodytes troglodytes,* 5 *P. troglodytes schweinfurthii*, 4 *P. troglodytes verus*, and 1 *P. troglodytes hybrid*), 20 gorillas (18 *Gorilla gorilla gorilla* and 2 *G. beringei beringei*), and 20 orangutans (19 *Pongo pygmaeus* and 1 *Pongo abelii*) were used for the analysis. Human specimens were collected at the University Museum, University of Tokyo. Wild specimens of 10 chimpanzees, 10 gorillas and 14 orangutans were obtained from MorphoSource (https://www.morphosource.org, Media ID: 3331; 3334; 101646; 101767; 102043; 102410; 102456; 102677; 103565; 103571; 3309; 3312; 3315; 4104; 84184; 84193; 101760; 102189; 102200; 103581; 3336; 3344; 3347; 3348; 102419; 102476; 102582; 102686; 102881; 102890; 102893; 103077; 103125; 103426). Specimens of seven chimpanzees, five gorillas, and four orangutans were captive cadaver feet donated to the Primate Research Institute, Kyoto University from zoos in Japan. Three dry bone specimens of chimpanzees were of wild individuals from the Mahale Mountain National Park, Tanzania. Five dry bone specimens of gorillas were of captive specimens housed at the National Museum of Nature and Science, Japan. Two dry bone specimens of orangutans were collected at the Laboratory of Physical Anthropology, Kyoto University. Tali from seven Japanese macaques (*Macaca fuscata*) were also included in this analysis as an outgroup. They were captive dry bone specimens housed at the Laboratory of Physical Anthropology, Kyoto University, except for one cadaver specimen studied in Ogihara et al.^15^. All samples were adult and free of obvious pathology. All tali were segmented from the CT scans, and bone models of the tali were generated in Mimics 22.0 (Materialise Inc., Leuven, Belgium). The left tali were mirrored and analyzed as right-sided specimens. Pixel size and slice interval of the CT scans were 0.16 mm for humans, ≤ 1.0 mm for chimpanzees, gorillas and orangutans, and ≤ 0.2 mm for Japanese macaques.

### Cone frustum approximation

**Figure 1:**
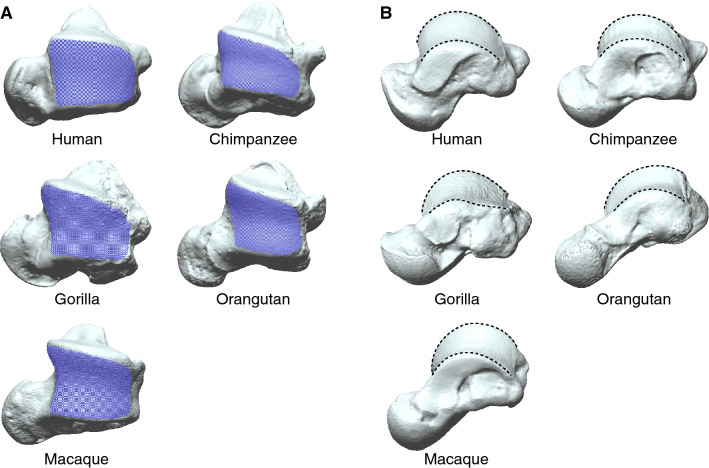
Three-dimensional models of the human, great ape, and macaque tali. (**A**) Superior view of the extracted trochlea articular surfaces. (**B**) Superomedial view of the medial and lateral rims. The medial and lateral rims of the talar trochlea in the human, chimpanzee, orangutan, and Japanese macaque are uniformly convex, but the medial rim of the gorilla is flattened.

**Figure 2:**
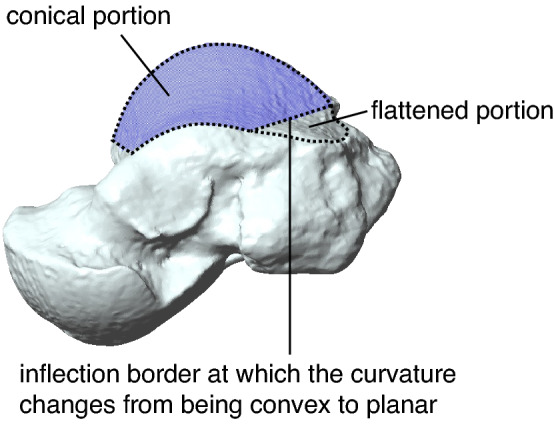
Superomedial view of the talar trochlea in the gorilla. The gorilla’s trochlea can be divided into the conical and flattened portions. The inflection border at which the curvature changes from being convex to planar was manually determined, and the conical portion of the articular surface was extracted.

The talar trochlea surface was extracted manually by outlining the visible border of the subchondral bone surface (Fig. 1A) using commercial software (Geomagic Design X, version 2019.0.3, 3D Systems Inc., Rock Hill, SC, USA). The medial malleolar extension anterior to the anterior border of the articular surface was not included to capture only the conical portion of the surface. Gorilla trochleae were observed to be quite unique in shape, and the posteromedial region of the talar trochlea was more planar and flattened compared to that of other species (Fig. 1B). Therefore, we extracted the gorilla trochleae in two ways: one including the whole trochlear surface and the other including only the conical portion of the surface but excluding the flattened posteromedial region of the trochlea (Fig. 2).

**Figure 3:**
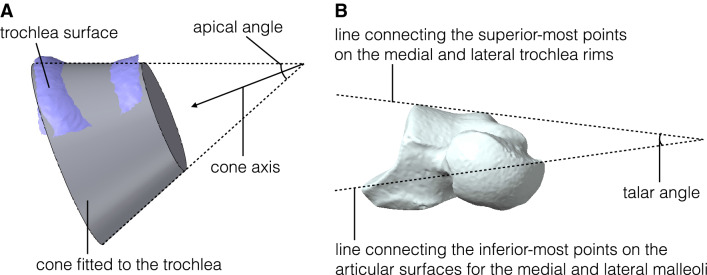
(**A**) Cone frustum approximation of the trochlea surface. The position and orientation of the cone axis and the apical angle were calculated so as to best fit the trochlea surface. (**B**) Definition of the talar angle in DeSilva^3^. The tali were placed so that the plantar edges of the calcaneal facets and the inferior aspect of the talar head were same plane. In this orientation, the angle between the line connecting the superior-most points on the medial and lateral trochlea rims and the line connecting the inferior-most points on the articular surfaces for the medial and lateral malleoli was measured.

The surface was then fitted by a cone frustum by minimizing the sum of the squared distances between a set of points comprising the talar trochlea and the cone frustum surface using the Solid Primitive command in Geomagic Design X (3D Systems Inc., Rock Hill, SC, USA) (Fig. 3A). The fitted cone surface was represented by the position and orientation of the cone axis and apical angle. The apical angle of the cone was positive if the apex of the cone was located on the medial side (Fig. 3A).

To evaluate how well the talar trochlea can be approximated by a cone frustum, the accuracy and precision of the surface approximation were evaluated for each specimen by calculating the mean and standard deviation of the differences between the points comprising the articular surface and the cone frustum surface using Geomagic XOS (version 5.0.0.0, 3D Systems Inc., Rock Hill, SC, USA).

To investigate the potential correspondence between the calculated apical angle and the talar angle^2,3,7^, each talus was placed in a 3D space so that the plantar edge of the calcaneal facet and the inferior aspect of the talar head were on the same plane, and the medial and lateral malleolar facets were in the same coronal plane^3^. The angle between the line connecting the superior-most points on the medial and lateral trochlear rims and the line connecting the inferior-most points on the articular surfaces of the medial and lateral malleoli in the coronal plane was measured as the talar angle (Fig. 3B).

### Three-dimensional geometric morphometrics

**Figure 4:**
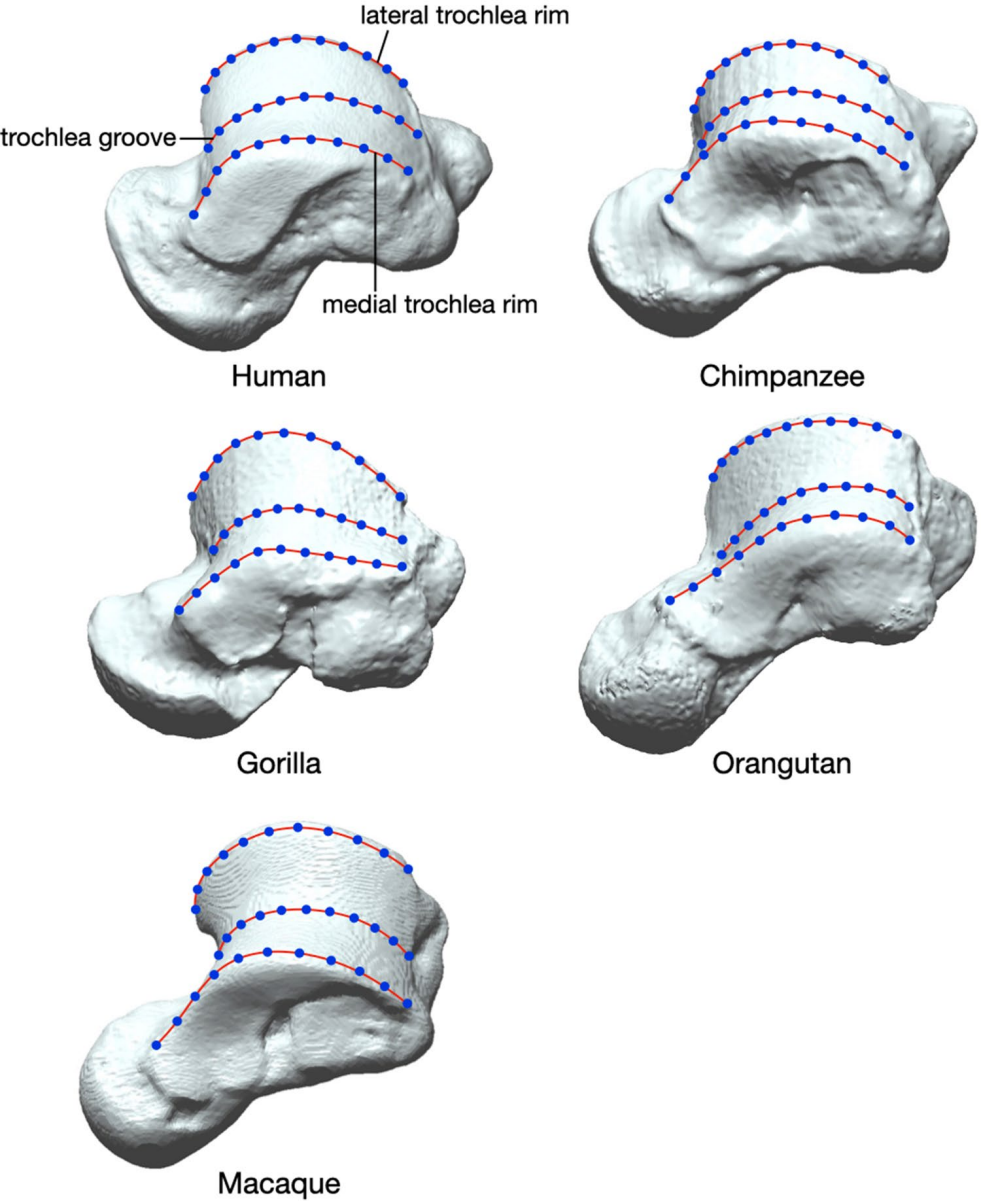
The medial and lateral trochlea rims and the trochlea groove (red curves) and the equally spaced points along the curves (blue points) to analyze the shape variations of the trochlea surface.

The medial and lateral rims and the central groove of the trochlea were approximated by a fifth-order Bezier curve using custom-made software^16^. Eleven equally spaced points along each of the curves were calculated (Fig. 4), and the coordinates of 33 landmarks were analyzed using geometric morphometrics^17–19^. The coordinates of the points were normalized by centroid size and registered using the Generalized Procrustes Analysis^20–22^ to remove variance associated with size, translation, and orientation. Principal component analysis was then conducted with the variance–covariance matrix of the Procrustes residuals to obtain the principal components (PCs) of shape variations among specimens.

### Statistical analyses

The apical angle of the cone and the PC scores were compared among species using analysis of variance (ANOVA). If ANOVA was significant, a post-hoc Tukey’s HSD test was performed. The Kruskal–Wallis test with a post-hoc Wilcoxon rank-sum test for multiple comparisons, followed by Bonferroni correction with the adjusted *P*-value set at *P* < 0.005 (0.05/10), was used if tests for normality or homogeneity of variance failed. Normality and homogeneity of variance were tested using the Shapiro–Wilk test and the Bartlett’s test, respectively. To test for significant difference between the apical and talar angles in each species a paired *t*-test was performed. The correlation between these angles was analyzed using Spearman’s rank correlation coefficient. The statistical significance level was set at *P*-value < 0.05. Data processing and analyses were implemented in the open source R software, version 3.5.2^23^ using the R package “geomorph”.

Both wild and captive specimens were included in the present study. To investigate the possible differences in the trochlea morphology between wild and captive specimens of the same species, we tested if the mean PC scores were statistically different between wild and captive specimens in chimpanzees, gorillas and orangutans using two-tailed *t*-test or Wilcoxon rank-sum test.

## Results

**Figure 5:**
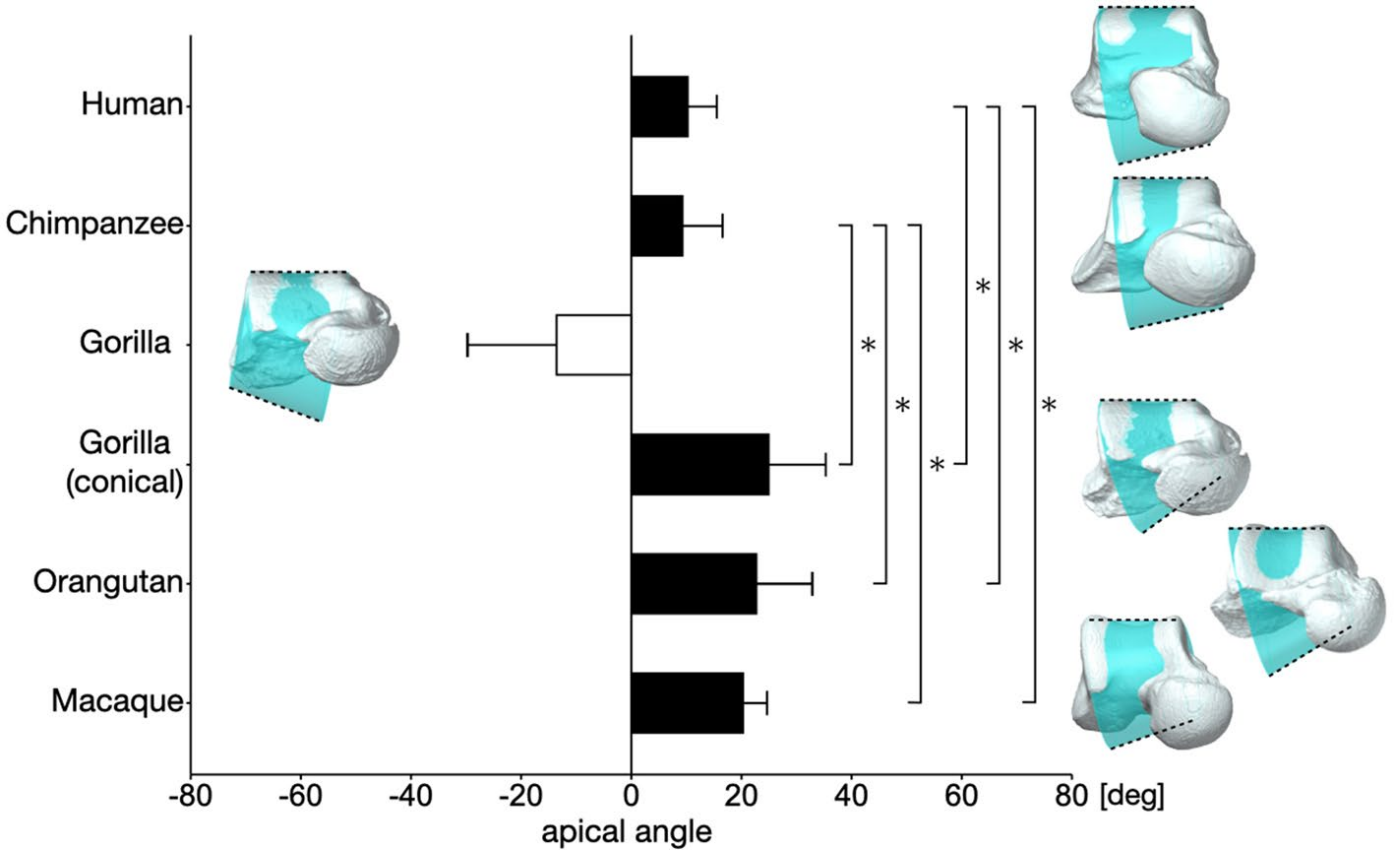
Cone frustum approximation of the talar trochlea surfaces, and comparison of the mean apical angles among humans, great apes, and macaques. The error bars indicate standard deviations. If the whole surface was approximated, the apical angle in gorillas was calculated to be negative, an indication that the apex was located on the lateral side of the talus. However, if only the conical portion was used for approximation, the apical angle was calculated to be positive like in other species. The asterisk indicates that there was a significant difference in the mean of the apical angle (*P* < 0.005).

**Figure 6:**
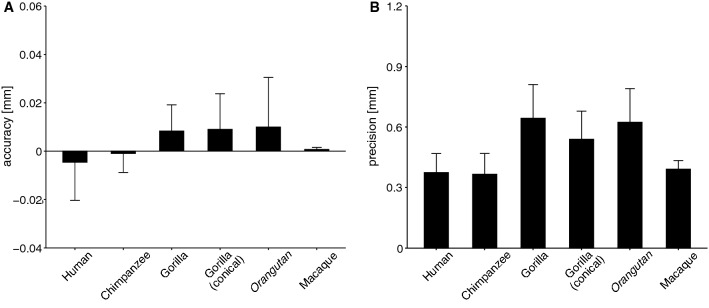
Accuracy (**A**) and precision (**B**) of the cone frustum approximation of the trochlea. Error bars indicate standard deviation.

The cone frustum surfaces that approximated the trochlear articular surfaces of each representative human, chimpanzee, gorilla, orangutan, and macaque tali, along with the calculated apical angles, are presented in Fig. 5. In gorillas, if the whole surface was used to approximate the trochlea surface, the apical angles were calculated as negative. However, if only the conical portion was used (Fig. 2), the apical angles were positive, as in other species. Errors associated with the approximation, that is accuracies and precisions, were lower than 0.07 mm and 1.2 mm, respectively, suggesting that the trochlear surfaces were successfully approximated by a cone frustum surface (Fig. 6). The errors tended to be larger for gorillas and orangutans.

Figure 5 shows that the apical angles were larger in gorillas and orangutans than in humans and chimpanzees. Kruskal–Wallis test showed the significant differences in the apical angle among the five species (*x*^2^ = 36.7, *P* < 0.0001). The apical angle of gorillas was found to be significantly larger than that of humans (*P* < 0.0001) and chimpanzees (*P* < 0.0001). The apical angle of orangutans was also significantly larger than that of humans (*P* = 0.0002) and chimpanzees (*P* < 0.0001). In addition, the apical angle of Japanese macaques was significantly larger than that of humans (*P* = 0.0003) and chimpanzees (*P* = 0.0007). However, no statistical difference was detected between the apical angles of humans and chimpanzees.

**Figure 7:**
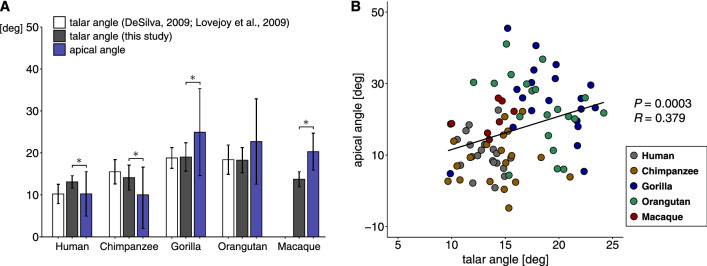
Comparison of the talar and apical angles among humans, great apes, and macaques (**A**). Significant differences between the apical and talar angles were detected in humans, chimpanzees, gorillas and Japanese macaques (^*^P < 0.05). A bivariate plot of the apical angle relative to the talar angle (**B**). There was a significant correlation between the apical and talar angles (*P* = 0.0003; *R* = 0.379).

Figure 7A compares the apical and talar angles of the five species. The talar angles calculated based on the present samples were generally in agreement with the values reported in the literature^3,7^. However, the apical angles were significantly smaller than the talar angles in humans (*P* = 0.04) and chimpanzees (*P* = 0.01) while those in gorillas and Japanese macaques were significantly larger than the talar angles (*P* = 0.03, *P* = 0.005, respectively) (Fig. 7A). Figure 7B shows a scatter plot of apical and talar angles. The graph indicates that the intraspecific variations in the angles were quite large. The correlation between the two angles was statistically significant (*P* = 0.0003), but the correlation coefficient was not large (*R* = 0.379), indicating that the talar angle did not clearly correspond with the apical angle.

**Figure 8:**
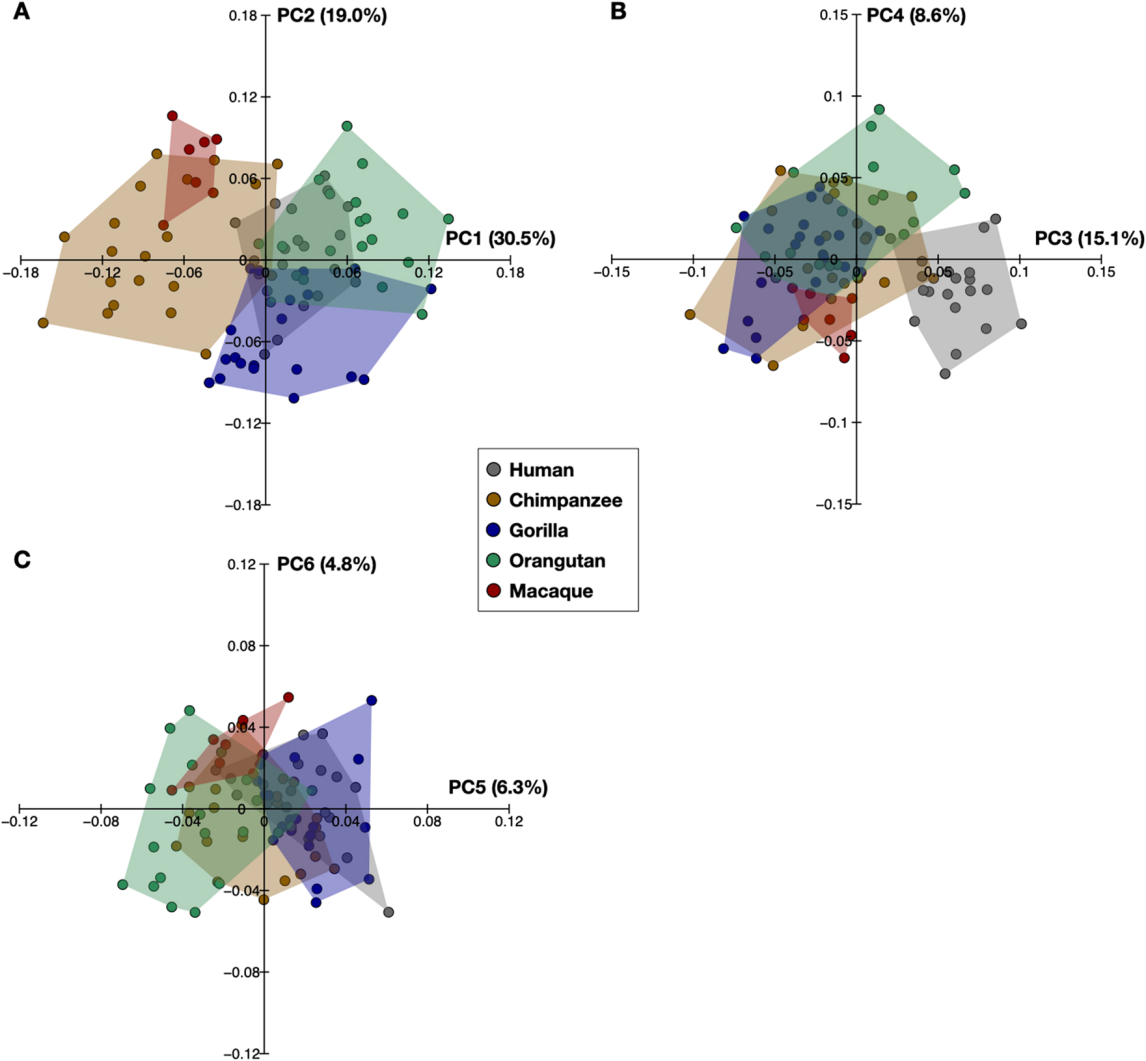
Scatter plot of the principal component (PC) scores. PC 1 versus PC 2 (**A**). PC 3 versus PC 4 (**B**). PC 5 versus PC 6 (**C**). The percentage of variance explained by each PC score is shown in parentheses.

The results of the geometric morphometric analysis of the trochleae are presented in Fig. 8 as a plot of PC1 versus PC2, PC3 versus PC4, and PC5 versus PC6. Based on a threshold of 5% variance explained, the first five PCs were considered dominant in the analysis. The first five PCs accounted for 79.5% of the total variance. We confirmed that there were no statistically significant differences in the PC scores between the wild and captive specimens, except for the PC4 score of chimpanzees and the PC3 and 4 scores of orangutans (Supplementary Information), indicating that the use of captive specimens has no major effect on the results of the present analysis.

**Figure 9:**
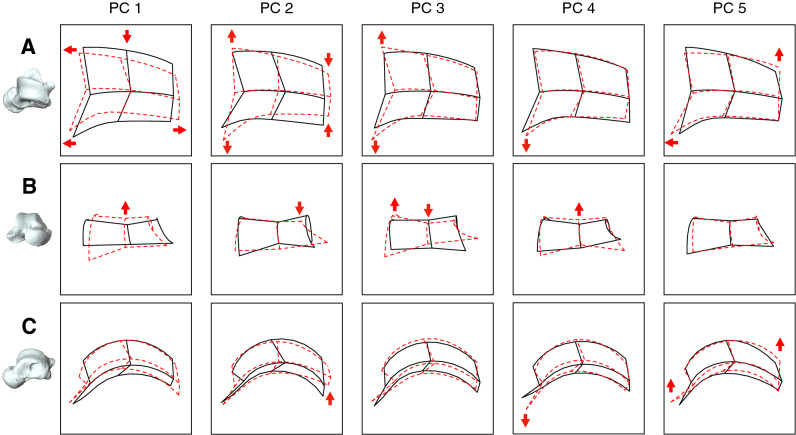
Shape variations represented by PC 1, PC 2, PC 3, PC 4, and PC 5. Shape variations are visualized with three-dimensional deformations of wireframe connecting points on each of the curves of the trochlea. Black line: PC 1 = 0.12, PC 2 = 0.10, PC 3 = 0.08, PC 4 = 0.06, and PC 5 = 0.05. Red line: PC 1 =  − 0.12, PC 2 =  − 0.10, PC 3 =  − 0.08, PC 4 =  − 0.06, and PC 5 =  − 0.05. Red arrows indicate characteristics of the negative score. The superior (**A**), anterior (**B**), and superomedial (**C**) views were presented.

ANOVA and the Kruskal–Wallis test identified significant differences in the trochlea shape among species for PC1 (*x*^2^ = 57.4, *P* < 0.0001), PC2 (*x*^2^ = 39.2, *P* < 0.0001), PC3 (*x*^2^ = 48.0, *P* < 0.0001), PC4 (*F* = 8.4, *P* < 0.0001), and PC5 (*x*^2^ = 36.2, *P* < 0.0001). The pattern of shape variations in Fig. 8 shows that the scores of PC1 in chimpanzees and Japanese macaques were significantly smaller than those of other non-human great apes and humans (Fig. 8A, Table 1). With decreasing PC1, the length and height of the trochlea increased while the width decreased (Fig. 9A,B). The curvature radii of the trochlea increased with decreasing PC1 (Fig. 9C).

**Table 1:** *P*-values of post-hoc multiple comparisons of PC scores among species.

	PC1	PC2	PC3	PC4	PC5
*Human* vs *Chimpanzee*	** < 0.0001**	0.799	** < 0.0001**	**0.045**	0.007
*Human* vs *Gorilla*	0.142	** < 0.0001**	** < 0.0001**	0.329	0.904
*Human* vs *Orangutan*	**0.0009**	0.253	** < 0.0001**	**0.0001**	** < 0.0001**
*Human* vs *Macaque*	** < 0.0001**	**0.0002**	** < 0.0001**	0.709	**0.001**
*Chimpanzee* vs *Gorilla*	** < 0.0001**	** < 0.0001**	0.015	0.882	0.013
*Chimpanzee* vs *Orangutan*	** < 0.0001**	0.429	0.398	0.349	0.005
*Chimpanzee* vs *Macaque*	0.081	**0.002**	0.646	**0.012**	0.288
*Gorilla* vs *Orangutan*	**0.0003**	** < 0.0001**	0.007	**0.0496**	** < 0.0001**
*Gorilla* vs *Macaque*	** < 0.0001**	** < 0.0001**	0.162	0.073	**0.0001**
*Orangutan* vs *Macaque*	** < 0.0001**	**0.003**	0.314	**0.0001**	0.198

*P*-values < 0.05 or < 0.005 (0.05/10) are in bold to indicate significant differences.

The gorillas had significantly lower PC2 scores than the other species (Fig. 8A, Table 1). With decreasing PC2, the anterior width of the trochlea increased, while the posterior width decreased (Fig. 9A), indicating a more trapezoidal shape of the trochlea in gorillas. The superior edge of the medial trochlea rim was shifted inferiorly with decreasing PC2 (Fig. 9B), making the superior surface of the trochlea face more medially in gorillas. The posteromedial point of the trochlea was shifted dorsally with decreasing PC2 (Fig. 9C).

The PC3 scores in great apes were significantly lower than those in humans (Fig. 8B, Table 1). With decreasing PC3, the most superior point of the middle trochlea was shifted inferiorly, while the lateral trochlea rim was shifted dorsally (Fig. 9B), indicating that the trochlear groove was deeper in great apes than humans.

Japanese macaques exhibited significantly smaller PC4 scores than chimpanzees and orangutans (Fig. 8B, Table 1). The anteromedial point of the trochlea was shifted medially (Fig. 9A) and inferiorly (Fig. 9C), and the height of the trochlea were slightly higher with decreasing PC4 (Fig. 9B).

The PC5 score in orangutans was significantly lower than that in gorillas and humans (Fig. 8C, Table 1). With decreasing PC5, the anteromedial and posterolateral points of the trochlea shifted anteriorly and laterally, respectively, representing more quadrangular trochlea shapes in orangutans (Fig. 9A). These points also shifted dorsally with decreasing PC5, indicating that the trochlear surface was relatively flatter in orangutans (Fig. 9C).

## Discussion

It is generally accepted that the trochlear surface of humans and great apes can be approximated by a cone frustum^1,2^, but no previous studies have demonstrated this, to the best of our knowledge. The present study demonstrated, for the first time, that the talar trochlea surfaces in humans and great apes can actually be well approximated by a cone frustum, as suggested by Inman^1^ and Latimer et al.^2^. However, in gorillas, if the whole region of the talar trochlea was approximated by the cone, the apex of the cone was found to be located on the lateral side of the trochlea, owing to the fact that the curvature radii of the medial rims of the gorilla trochlea were larger than those of the lateral rims because the surface of the posteromedial portion of the trochlea was flattened, as shown in Fig. 2, as previously reported^12,24^. To bring the apex of the cone on the medial side of the trochlea as in other species, only the conical portion of the trochlea should be used to approximate the trochlear surface in gorillas.

Although the calculated apical angles of the approximated cones were significantly correlated with the talar angles (*P* = 0.0003) conventionally used to estimate the apical angles of the cones^3,7^ (Fig. 7), the present study demonstrated that the correlation between these angles was weak (*R* = 0.379). Geometrically, the talar angle should be half of the apical angle because the cone apex is at the intersection between the line passing through the supratalar surface and the talocrural rotational axis corresponding to the cone axis, and the talar angle is the angle between the two lines on the coronal plane. However, this geometrical relationship was not clearly observed in our study (Fig. 7B). These discrepancies indicate that the talar angle could not precisely estimate the apical angle of the cone frustum fitted to the trochlea. This is because the apical angle of the cone is a 3D quantity but the talar angle estimated the apical angle only two-dimensionally on the coronal plane, and the angle projected on the transverse plane was not incorporated. Therefore, the talar angle cannot be used as a synonym of the apical angle of the cone frustum approximating the trochlea but is only a 2D angle of the trochlear rotation axis estimated based on the two inferior-most points of the tibial and fibular facets with respect to the superior surface of the talar trochlea.

The present study demonstrated statistically significant interspecific differences in the apical angle. The apical angles of the humans and chimpanzees were significantly smaller than those of gorillas and orangutans, but no statistical difference was detected between humans and chimpanzees, as well as between gorillas and orangutans (Fig. 5). This result contradicted the findings of Latimer et al.^2^ and DeSilva^3^, who reported that the talar angle of humans was smaller than that of chimpanzees and gorillas. In the present study, the human talar angle was confirmed to be significantly smaller than that of gorillas (*P* < 0.0001), but not that of chimpanzees (*P* = 0.149). These findings suggest that the talar angle may not be as different as once thought between humans and chimpanzees. Humans engage in habitual bipedalism. Gorillas engage in knuckle-walking and are regarded as the most terrestrial of the great apes, although western lowland gorillas are known to climb on trees for feeding to some extent^25,26^. Chimpanzees also engage in knuckle-walking and travel between feeding trees mainly on the ground^27^, but they frequently engage in vertical climbing and suspensory locomotion as well^28,29^. Orangutans are fundamentally quadrumanous climbers in the rain forest canopy, and they seldom walk on the ground^30–32^. Therefore, there is a distinctive difference in the degree of arboreality among species. However, the present study suggested that the apical (or talar) angle is not clearly associated with the degree of arboreality in humans and great apes.

Our geometric morphometric analysis clearly extracted and visualized interspecific differences in the shape of the talar trochlea among humans and great apes, which were not clearly observed in the comparisons of the apical and talar angles. Chimpanzees, along with macaques, possessed a longer and highly curved talar trochlea (Fig. 9A). The longer and curved trochlea possibly allows greater sagittal rotation of the tibia on the trochlea surface at the ankle joint, possibly increasing the mobility of the ankle plantar and dorsiflexion. The greater mobility in dorsiflexion of the ankle joint has been suggested to facilitate vertical climbing^3,33,34^. It was also found that chimpanzees and gorillas possessed more trapezoidal trochleae than humans (Fig. 9A) as reported by previous studies^5,12,24^. Because the anterior region of the superior surface of the talar trochlea contacts the tibial plafond during dorsiflexion^35^, the relatively wider anterior part of the trochlea may increase the contact area of the ankle joint during ankle dorsiflexion, possibly to adapt to greater weight bearing when the ankle is in a dorsiflexed posture. Conversely, the human (and macaque) trochleae did not exhibit such a feature, but the trochlea was more rectangular than those of the other species, possibly to adapt to increased contact force during plantarflexion, particularly in the late stance phase of human walking^36^. The gorilla trochlea differed from that of the other four species in having a less-curved posteromedial trochlea, more medially projected medial malleolar extension onto the talar neck, and deeper central groove. The former two features may be related to the reduced range of the talocrural joint in gorillas to accommodate their large body mass^37,38^. The enhanced central groove provides increased stability of the talocrural joint in the mediolateral direction. The orangutan trochlea is unique in having a wider posterior margin of the trochlea and a more dorsally turned anteromedial and posterolateral trochlear surface, indicating that the trochlear surface is relatively flatter. The functional significance of this morphological feature is obscure, but it might be related to the fact that the orangutan foot functions as a suspensory supporting organ for hook-like digital gripping without involvement of the hallux, although in the chimpanzee and gorillas, the foot may be adapted to hallux-assisted power gripping^39^. However, to make further inferences about the form-function relationship of the talar trochlea, data on actual foot use in African great apes and orangutans during terrestrial and arboreal locomotion are lacking and should be investigated in future studies.

The present study demonstrated that the talar trochlea was clearly different in shape between humans and great apes. However, the present study also found that the talar shape was not clearly associated with the differences in locomotor behavior and the degree of arboreality among the species. For example, the apical or talar angle of the trochlea is believed to be correlated with the degree of foot inversion facilitating vertical climbing by positioning the foot sole against the tree substrate^2,3^, but the apical angle of the trochlea was not substantially different between humans and chimpanzees (Fig. 5). In addition, the scatter diagram (Fig. 8A) demonstrated that the trochlea is more similar in shape between humans and orangutans, which differ substantially in locomotor behavior. The morphology of the talar trochlea may not be a distinct skeletal correlate of locomotor behavior possibly because the talar morphology is determined not only by locomotor behavior, but also by other factors such as phylogeny and body size. This is consistent with Sorrentino et al.^5^ indicating that the morphology of the hominin trochlea is not unequivocally linked to locomotor behavior. Therefore, caution needs to be exercised in assessing the morphological affinities of fossil hominid tali to reconstruct their locomotor behavior.

The present study has some limitations. First, the apical angle of the cone may be affected by the manual extraction of the trochlear surface. However, we defined the extracted region as objectively as possible; hence, this effect was confirmed to be relatively minor. Second, the present study included both wild and captive specimens in non-human species. However, we confirmed that the use of captive specimens has only minor effect on our results (Supplementary Information). Third, the present study did not investigate morphological variations in the distal tibia^40^ and fibula^41^, which are also important determinants of the mobility of the talocrural joint.

In conclusion, we demonstrated that the trochlea of the talus can be approximated by a conical surface in humans and great apes. However, it was found that the calculated apical angle did not clearly correspond to the degree of arboreality. Our detailed trochlear shape analysis using geometric morphometrics successfully extracted interspecific differences in the morphology of the trochlea; however, no clear association was observed between the morphology and locomotor behavior. The morphology of the talar trochlea may not be a distinct skeletal correlate of locomotor behavior.

## Supplementary Information

Supplementary Information.

## Data Availability

The data that support the findings of this study are available from the corresponding authors upon reasonable request.
