# Supplementary material for: Talar trochlear morphology may not be a good skeletal indicator of locomotor behavior in humans and great apes
Source: Sci Rep. 2021 Dec 15;11:24063. doi: 10.1038/s41598-021-03429-y (PMC8674354; doi:10.1038/s41598-021-03429-y)
Supplement: Supplementary file 1 — Supplementary Information. [file 41598_2021_3429_MOESM1_ESM.pdf]

Supplementary Information

Talar trochlear morphology may not be a good skeletal indicator of locomotor behavior in humans and great apes

Shuhei Nozaki[1], Motoharu Oishi[2], and Naomichi Ogihara[1]

[1]Laboratory of Human Evolutionary Biomechanics, Department of Biological Sciences, Graduate School of Science, The University of Tokyo, Tokyo, 113-0033, Japan.

[2]Laboratory of Anatomy, School of Veterinary Medicine, Azabu University, Sagamihara, Kanagawa, 252-5201, Japan.

Supplementary Table. *P*-value of statistical tests (two-tailed *t*-test or Wilcoxon rank-sum test) to compare between the wild and captive specimens in great apes

species	PC1	PC2	PC3	PC4	PC5
Chimpanzee	0.198	0.311	0.757	**0.005**	0.702
Gorilla	0.136	0.631	0.481	0.971	0.711
Orangutan	0.049	0.841	**0.012**	**0.015**	0.919

P-values < 0.017 (0.05/3) are in bold to indicate significant differences.

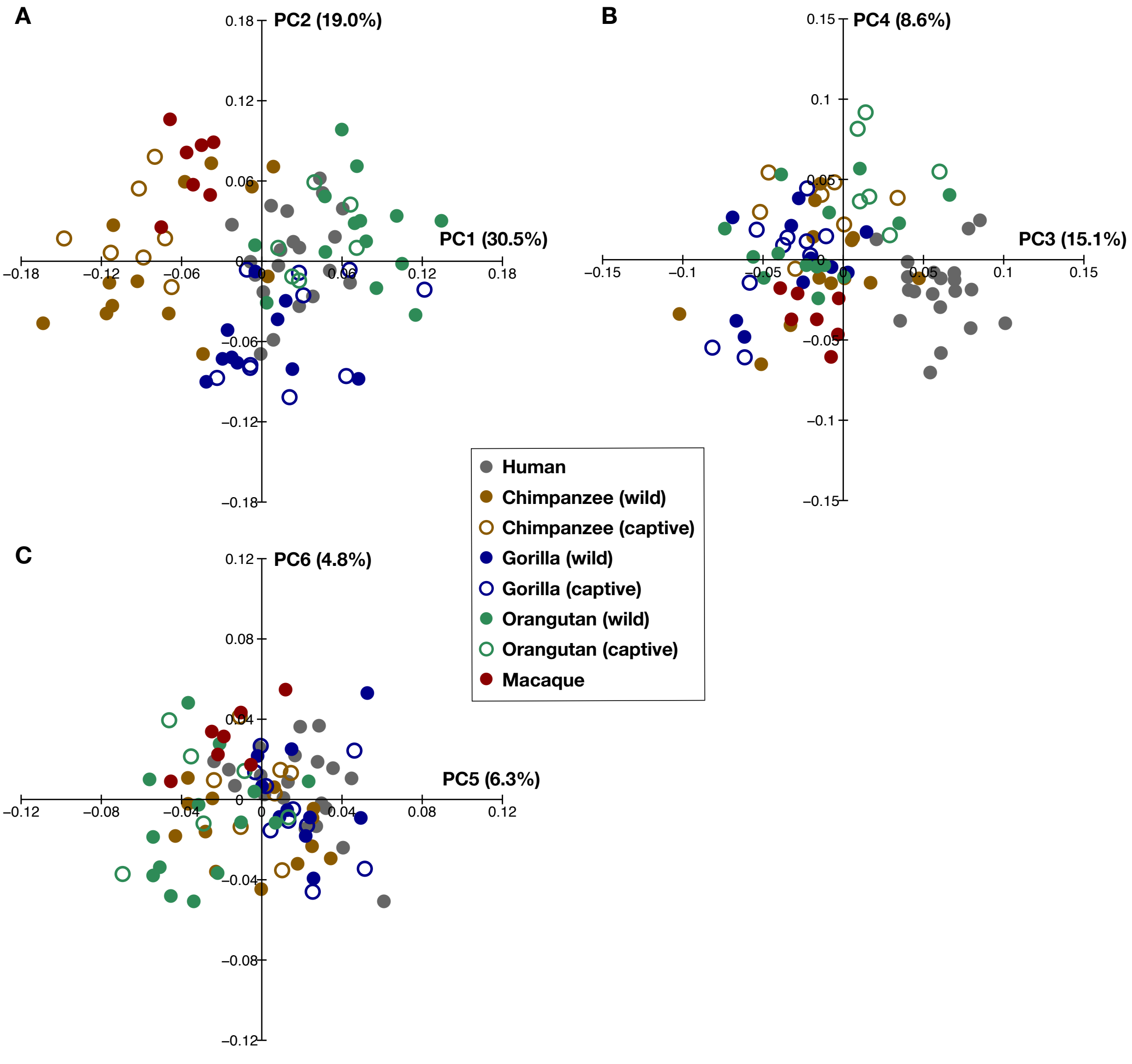

Supplementary Figure. Scatter plots of the PC1 versus PC2 (**A**), PC3 versus PC4 (**B**) and PC5 versus PC6 (**C**) in geometric morphometric analysis (wild =solid circle, captive = open circle). The percentage of variance explained by each PC score is shown in parentheses.
